# In Situ Growth of Metal–Organic Frameworks (MOFs) Within Porous Silicon Carbide (p-SiC) for Constructing Hierarchical Porous Composites

**DOI:** 10.3390/nano16020117

**Published:** 2026-01-15

**Authors:** Long Zhou, Guangzhi Liao, Tingting Lin, Wensong Huang, Jiawei Zhang, Ruiqi Fan, Yanghui Li, Xiaolin Zhang, Ziyun Cheng, Lizhi Xiao

**Affiliations:** 1 State Key Laboratory of Petroleum Resources and Engineering, China University of Petroleum (Beijing), Beijing 102249, China; 2 College of Instrumentation and Electrical Engineering, Jilin University, Changchun 130026, China; 3 Research Institute of Petroleum Exploration and Development, PetroChina, Beijing 100083, China

**Keywords:** metal–organic frameworks (MOFs), porous silicon carbide (p-SiC), composites, rock-analog porous media, CO_2_ adsorption

## Abstract

Metal–organic frameworks (MOFs) typically exist in the form of powders or dispersed crystals, which limits their direct application in practical engineering scenarios that require monolithic structures and processability. To address this issue, the present study successfully anchored MOF (zeolitic imidazolate framework-8, ZIF-8) nanocrystals within a porous silicon carbide (p-SiC) substrate via a facile in situ growth strategy, achieving both stable macroscopic loading and intimate microscopic interfacial bonding. The resulting ZIF-8/p-SiC composite exhibits a hierarchical porous structure, with a specific surface area approximately 183 times higher than that of the raw p-SiC, alongside a substantially enhanced CO_2_ adsorption capacity. By utilizing a low-cost p-SiC support and mild ZIF-8 synthesis conditions, this work demonstrates excellent reproducibility and scalability, providing a facile and effective pathway for fabricating MOF/porous media composite systems that possess both superior mechanical properties and tailored pore structures. Additionally, the developed MOF/p-SiC composites can serve as controllable rock-analog porous media, offering new perspectives for investigating MOF-rock interfacial interactions and CO_2_ geological sequestration mechanisms, thereby establishing an organic link between fundamental materials science and geological engineering applications.

## 1. Introduction

Metal–organic frameworks (MOFs), also referred to as porous coordination polymers (PCPs), are a class of porous crystalline framework materials synthesized via the self-assembly of ordered metal ion nodes or clusters with organic ligand units [1,2]. In recent years, by capitalizing on intermolecular interactions and metal-ligand coordination, researchers have designed a diverse array of two- and three-dimensional metal–organic networks possessing high porosity, as well as superior ion exchange and adsorption capabilities [3,4]. These materials find extensive applications across various fields, including gas storage and separation [5], carbon capture and conversion [6], heterogeneous catalysis [7], sensing [8], and biomedicine [9].

However, MOFs typically exist in powder or discrete crystal form, which presents challenges in terms of stability, recyclability, and reusability, thereby restricting their further processing, transfer, handling, and practical implementation [10]. To address these limitations, interdisciplinary composite strategies for MOFs have emerged. Researchers have developed novel composites by supporting MOFs onto various porous substrates, producing materials such as MOF/polymer [11], MOF/clay [12], MOF/wood [13], and MOF/hydrogel composites [14]. While these composites successfully retain the intrinsic structure and porosity of MOF crystals, they often suffer from relatively weak mechanical properties or low permeability due to the well-known trade-off between high porosity and superior mechanical strength [15]. These drawbacks substantially limit their suitability for pressure-driven gas or liquid adsorption and separation processes [16].

Porous silicon carbide (p-SiC) is a highly promising support substrate for MOFs. Composed of a robust SiC framework, p-SiC possesses a unique combination of merits, including a tunable pore structure, superior mechanical strength, exceptional corrosion resistance, high tolerance to elevated temperatures and pressures, and versatile processability for shaping [17,18]. It provides abundant nucleation sites and transport channels, facilitating the uniform growth of MOFs throughout the entire scaffold. Furthermore, the active oxygen-containing functional groups on the p-SiC surface and the surface roughness [19] can promote heterogeneous nucleation and the stable anchoring of MOF crystals within the pore channels. To date, research leveraging these beneficial properties to develop MOF/p-SiC composites remains unexplored.

On the other hand, CO_2_ has been widely injected into subsurface reservoirs for geological sequestration and enhanced oil recovery (EOR) [20]. Reservoir components are typically rich in silicon (e.g., siliceous clastic rocks such as quartz sandstone) and carbon (e.g., carbonate rocks such as limestone and dolomite), and their physicochemical properties directly influence the adsorption, diffusion, and long-term stable sequestration processes of CO_2_ [21]. However, the complex and uncontrollable pore structures of natural rocks hinder the elucidation of CO_2_ capture mechanisms and the optimization of sequestration efficiency. As an artificial porous material constructed from Si–C bonds, p-SiC exhibits certain similarities to reservoir rocks in terms of composition and mechanical characteristics. Moreover, the hierarchical pore structure of MOF/p-SiC can simulate the organic and inorganic pores of rocks, and its surface chemical properties can be precisely tailored. Therefore, it can be regarded as a rock-analog porous media, providing an ideal medium for the quantitative investigation of CO_2_ adsorption and transport behaviors within rock pores.

**Figure 1:**
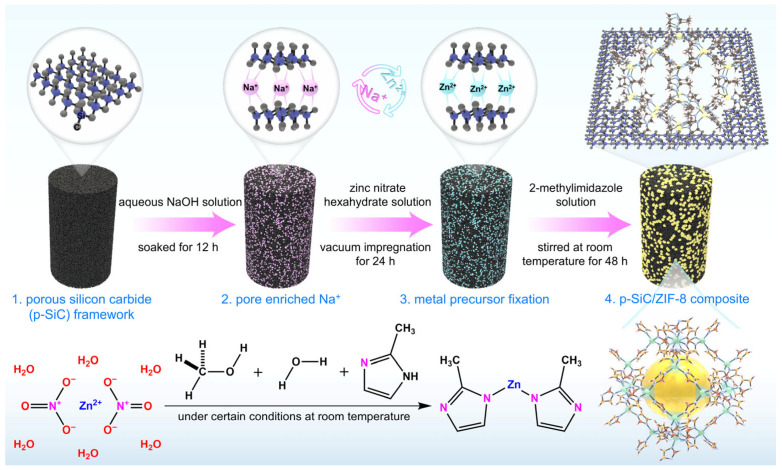
Schematic representation of the fabrication process to obtain ZIF-8/p-SiC composites.

Herein, this work proposes the in situ growth of MOF crystals within the pores of p-SiC to construct a MOF (zeolitic imidazolate framework-8, ZIF-8)/p-SiC composite featuring hierarchical pore structure, superior mechanical strength, and a high specific surface area (Figure 1). The MOF provides a micro- and mesoporous network for adsorption and molecular sieving, while the macroporous SiC framework serves as a robust mechanical support and transport channel. Consequently, this architecture offers a novel design strategy for developing functionalized MOF/porous media tailored for CO_2_ geological sequestration.

## 2. Experimental Section

### 2.1. Materials and Chemicals

p-SiC was customized into plunger-shaped samples with dimensions of 50 mm (*L*) × 25 mm (*Φ*), and a porosity of 30–35%. Zinc nitrate hexahydrate (Zn(NO_3_)_2_·6H_2_O, 99%), 2-methylimidazole (2-MeIm, C_4_H_6_N_2_, 99%), methanol (≥99.9%), sodium hydroxide (NaOH, 98%) and deionized water (DI) were purchased from Shanghai Macklin Biochemical Co., Ltd. (Shanghai, China). All chemicals were used as received.

### 2.2. Preparation of ZIF-8

ZIF-8 was synthesized according to the previously reported method [22]. A solution of 2.4 g of Zn(NO_3_)_2_·6H_2_O in 40 g of methanol and 3 g of DI and a second solution containing 6.6 g of 2-MeIm in the same solvent mixture were prepared. Both solutions were mixed and then stirred at room temperature for 48 h. The obtained solid was collected by centrifugation and thoroughly washed with methanol (3 times). The product was subsequently dried under vacuum overnight at 100 °C.

### 2.3. Preparation of ZIF-8/p-SiC Composites

The p-SiC was initially washed with DI and subsequently dried under vacuum at 100 °C for 48 h. The dried p-SiC was then soaked in a 15% (*w*/*v*) NaOH solution for 12 h to enrich Na^+^ on the surface of its pores, followed by multiple rinses with DI until the solution reached neutral pH. The pretreated p-SiC was immersed in a solution of Zn(NO_3_)_2_·6H_2_O (2.4 g) dissolved in methanol (40 g) and DI (3 g) to adsorb Zn^2+^ onto the pore surfaces of p-SiC. A vacuum impregnation was maintained at room temperature for 24 h to ensure sufficient ion exchange between Zn^2+^ and Na^+^ on the pore surfaces. Subsequently, a solution of 2-MeIm (6.6 g) in methanol (40 g) and DI (3 g) was added, and the mixture was stirred at room temperature for 48 h to form the MOF/p-SiC composite. The resulting material was washed three times with methanol for 60 min each to remove unreacted precursors and then dried under vacuum at 100 °C for 48 h to obtain the ZIF-8/p-SiC composite.

### 2.4. Characterizations

The morphology and microstructure of the samples were characterized using a field emission scanning electron microscope (FESEM, ZEISS GeminiSEM 300, Jena, Germany) equipped with an energy-dispersive X-ray spectroscopy (EDS, OXFORD SmartEDX, London, UK) system. Prior to imaging, all samples were coated with a 10 nm-thick Au layer using a metal sputter coater to enhance conductivity. Crystalline structures were analyzed by X-ray diffraction (XRD, D8 Advance, Bruker, Bremen, Germany) over a 2*θ* = 5–90° scan range, with an angular step size of 0.03° and a counting time of 1 s per step. Functional groups were identified using Fourier-transform infrared spectroscopy (FTIR, Thermo Fisher Scientific Nicolet iS20, Waltham, MA, USA) equipped with an ATR module, covering the spectral range of 400–4000 cm^−1^. The elemental composition and chemical states were analyzed using X-ray photoelectron spectroscopy (XPS, Thermo Scientific K-Alpha, Waltham, MA, USA). Zeta potential measurements were conducted using a Zeta potential analyzer (Zetasizer Nano ZS90, Malvern, UK). N_2_ adsorption–desorption experiments were performed at 77K using a specific surface area and pore size analyzer (JW-BK200C, JWGB, Beijing, China). Additionally, N_2_ and CO_2_ adsorption isotherms were recorded at 298 K using the same apparatus. Prior to gas adsorption measurements, all samples were degassed under vacuum at 100 °C for 12 h.

## 3. Results and Discussion

### 3.1. Discussion of System Selection

To ensure the mechanical stability of the MOF composites, the materials were selected based on the following considerations. p-SiC was chosen as the support substrate due to its abundance, economic viability, and sustainability. This material has been extensively utilized in applications such as high-temperature filters and catalyst supports [17]. Regarding the framework component, ZIF-8 was chosen as one of the most widely investigated MOF subclasses, offering diverse properties and applications. Composed of Zn^2+^ nodes and 2-methylimidazole linkers, ZIF-8 features a sodalite (SOD) topology characterized by uniform micropores and a high specific surface area. Furthermore, its mild and controllable synthesis conditions are conducive to industrial-scale production [23]. We anticipate that the combination of the robust p-SiC framework with the high specific surface area and porous functionality of ZIF-8 will endow the resulting composites with exceptional structural stability and versatile application potential.

### 3.2. Composition and Structure

Recent first-principles modeling studies on interfacial materials have indicated that interfacial bonding configurations play a pivotal role in determining the structural stability and functional performance of composites [24]. Consequently, prior to ZIF-8 loading, we subjected the p-SiC samples to a facile and effective NaOH solution pretreatment. This procedure served two primary functions: (i) the alkaline treatment introduced Na^+^ and hydroxyl (-OH) groups onto the SiC surface, facilitating ion exchange with Zn^2+^ from the precursor solution. This process promoted the local enrichment of metal ions, thereby generating abundant chemical nucleation sites favorable for ZIF-8 growth and ensuring uniform nucleation throughout the SiC framework; and (ii) the etching effect of NaOH increases the surface roughness of SiC, enhancing the anchoring of ZIF-8 on the SiC substrate. Zeta potential analysis shows that the negative surface charge of p-SiC samples increases from −25.30 ± 2.75 mV to −48.60 ± 1.20 mV after the pretreatment, resulting in a strong affinity for metal cations (Table 1).

**Figure 2:**
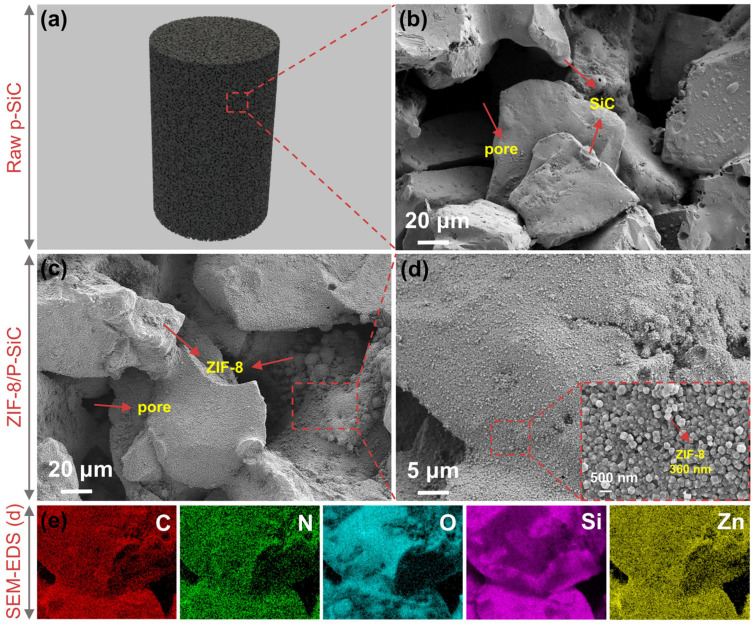
(**a**) Photograph of p-SiC. SEM images of (**b**) raw p-SiC and (**c**,**d**) ZIF-8/p-SiC composite; the inset in (**d**) shows a high-magnification SEM image of the ZIF-8 nanocrystals. (**e**) EDS elemental mapping of C, N, O, Si, and Zn distributions in the ZIF-8/p-SiC composite.

The subsequent step involved the in situ formation of ZIF-8 crystals within the pretreated p-SiC. SEM images illustrate the internal sectional microstructures of the raw p-SiC and the ZIF-8/p-SiC composite (Figure 2). The p-SiC consists of irregularly shaped SiC particles, with numerous interconnected pore channels formed between the particles. These channels are primarily created by the interparticle voids and are partitioned by dense SiC walls (Figure 2b). In addition, the morphology of the ZIF-8 crystals grown on the SiC pore surfaces is clearly visualized in Figure 2c. The ZIF-8 crystals exhibit a uniform particle size of approximately 360 nm on the pore surfaces of p-SiC, forming a compact deposition layer. These crystals exhibit a characteristic rhombic dodecahedral morphology (Figure 2d), which is consistent with the intrinsic crystal structure of ZIF-8 [25]. In contrast to the irregular agglomeration commonly observed in traditional bulk powder synthesis [26], the ZIF-8 crystals grown in situ on the p-SiC substrate demonstrate superior dispersity and well-defined crystal facets. The uniform distribution of ZIF-8 nanocrystals can be attributed to the abundant nucleation sites generated by the -OH groups introduced onto the p-SiC surface following NaOH pretreatment. These densely distributed nucleation centers promote homogeneous nucleation while suppressing uncontrolled crystal overgrowth during the growth process [13], thereby facilitating the formation of a uniform and densely packed MOF layer.

EDS elemental mapping of the ZIF-8/p-SiC composite reveals a uniform distribution of C, N, O, Si, and Zn elements across the pore surfaces (Figure 2e), where Zn originates exclusively from the ZIF-8 crystals. The mass percentage of Zn was determined to be approximately 3.21 wt% (Table 2). Based on this value, the ZIF-8 loading in the composite is estimated to be ~11.2 wt%. This relatively low loading aligns with the preservation of the macroscopic p-SiC framework integrity, indicating that ZIF-8 nanocrystals are predominantly anchored onto the pore surfaces rather than filling or blocking the macroscopic channels. Furthermore, following the synthesis of the ZIF-8/p-SiC composite, a sorbent activation procedure was conducted. It involves two steps: repeated washing with methanol, followed by heating under vacuum to fully remove the excess precursors that could otherwise block the microporous structure of ZIF-8.

**Figure 3:**
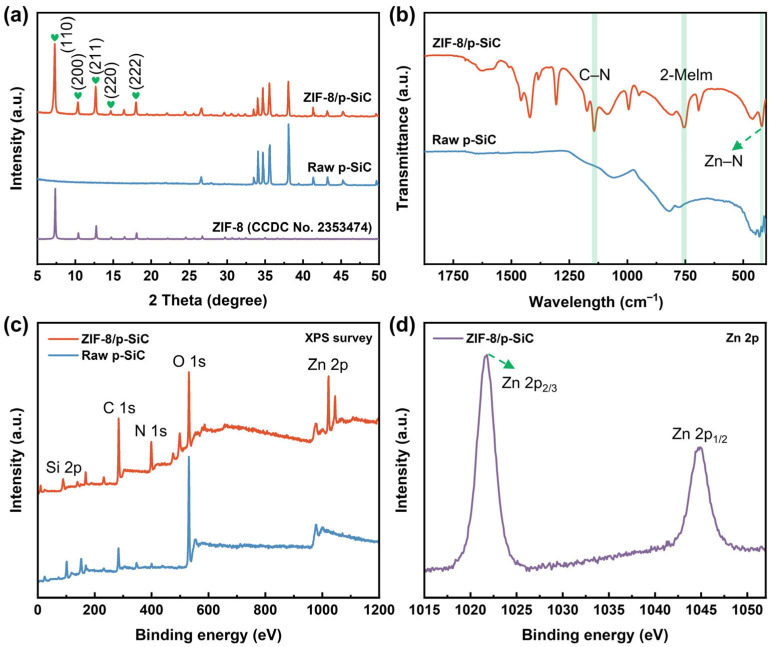
(**a**) XRD patterns of ZIF-8, raw p-SiC, and ZIF-8/p-SiC composite. (**b**) FTIR spectra of raw p-SiC and ZIF-8/p-SiC composite. (**c**) XPS survey spectra of raw p-SiC and ZIF-8/p-SiC composite. (**d**) High-resolution Zn 2p XPS spectrum of ZIF-8/p-SiC composite.

The XRD pattern of the ZIF-8/p-SiC composite exhibits diffraction peaks at 2*θ* = 7.3°, 10.3°, 12.7°, 14.7°, and 18.0°, corresponding to the (110), (200), (211), (220), and (222) crystal planes of ZIF-8, respectively (Figure 3a), which are consistent with the characteristic diffraction peaks reported for ZIF-8 in the literature [27]. The FTIR spectra exhibit characteristic absorption bands at 1143 cm^−1^, 755 cm^−1^, and 422 cm^−1^, corresponding to the C–N stretching of the imidazole ring, the out-of-plane bending vibration of the 2-MeIm linker, and the Zn–N coordination framework in ZIF-8, respectively (Figure 3b) [28]. Additionally, the XPS survey spectra confirm the presence of C, N, O, Si, and Zn elements in the ZIF-8/p-SiC composite (Figure 3c). The high-resolution Zn 2p spectrum of the composite is consistent with previously reported data for ZIF-8 (Figure 3d) [29]. Based on the aforementioned morphological and phase analyses, it can be concluded that ZIF-8 crystals have been successfully grown in situ within the p-SiC.

**Figure 4:**
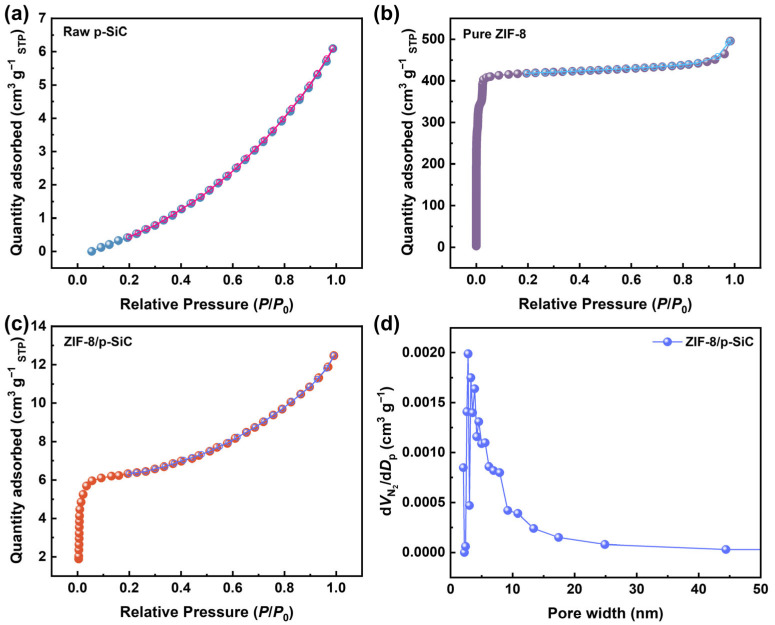
N_2_ adsorption and desorption isotherms of (**a**) raw p-SiC, (**b**) pure ZIF-8, and (**c**) ZIF-8/p-SiC composite. (**d**) BJH pore size distribution of ZIF-8/p-SiC composite.

To investigate the overall pore characteristics of the ZIF-8/p-SiC composite, N_2_ adsorption and desorption isotherms of raw p-SiC, pure ZIF-8, and the ZIF-8/p-SiC composite were measured at 77 K (Figure 4). The results indicate that the composite exhibits hybrid characteristics combining Type-I and Type-II isotherms. In the low-pressure region (*P*/*P*_0_ = 0–0.05), the ZIF-8/p-SiC composite displays a sharp N_2_ uptake (Figure 4c), a feature highly consistent with the adsorption behavior of pure ZIF-8 (Figure 4b), indicating the presence of ZIF-8-derived microporous structures in the composite. As *P*/*P*_0_ approaches 1, the adsorption isotherm shows an unstable and unsaturated trend similar to that of raw p-SiC (Figure 4a,c), suggesting the coexistence of macroporous structures. Therefore, the ZIF-8/p-SiC composite possesses a hierarchical pore structure composed of micropores originating from ZIF-8 and macropores inherited from the p-SiC scaffold. The corresponding pore parameters are shown in Table 3.

The uniform nanoscale dimensions of the grown ZIF-8 crystals are critical for enhancing adsorption performance. Compared to bulk crystals, the nano-size effect not only maximizes the contact sites at the gas–solid interface, thereby promoting initial adsorption kinetics, but also shortens the intracrystalline diffusion pathways [30], facilitating faster adsorption rates. This is correlated with the higher specific surface area of nanosized ZIF-8 (1588 m^2^ g^−1^) and the greater abundance of surface-active functional groups [31]. The ZIF-8/p-SiC composite shows a BET specific surface area of 18.3 m^2^ g^−1^, which is 183 times higher than that of raw p-SiC (0.1 m^2^ g^−1^), and a pore volume of 0.021 cm^3^ g^−1^. This substantial increase in specific surface area compared with raw p-SiC confirms the presence of ZIF-8 within the p-SiC. The corresponding BJH pore size distribution is shown in Figure 4d, yielding a median pore diameter of 3.28 nm for the ZIF-8/p-SiC composite.

### 3.3. Assessment of CO_2_ Adsorption Performance

**Figure 5:**
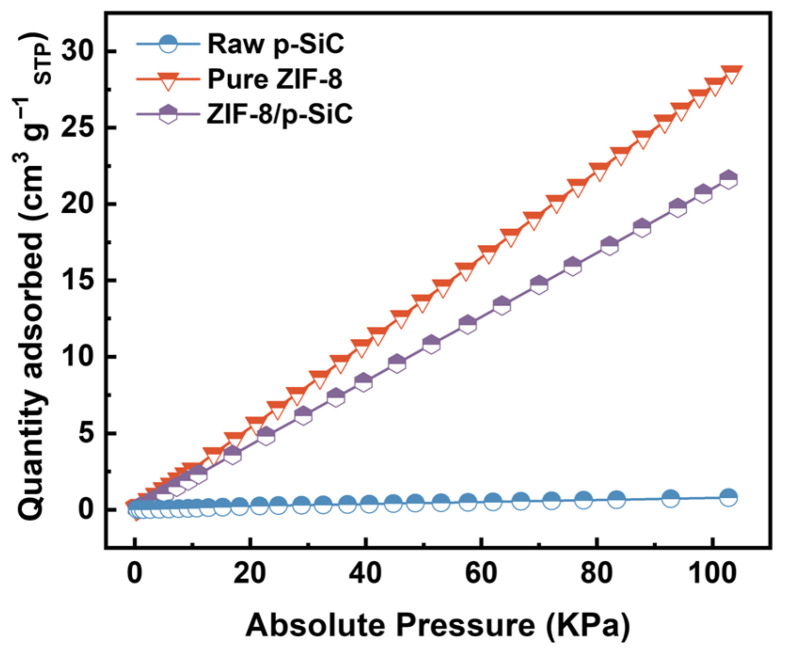
CO_2_ adsorption isotherms of raw p-SiC, pure ZIF-8, and ZIF-8/p-SiC composite at room temperature (298 K).

Such hierarchically organized porous materials are of high relevance for applications dealing with macromolecules and nanomaterials. The microporous network of the composite provides shape selectivity function for guest molecules such as CO_2_ and N_2_, while the meso- and macroporous networks enhance accessibility to active sites and facilitate diffusion kinetics. To validate the functionality of the ZIF-8/p-SiC composite, its CO_2_ adsorption capacity was evaluated at room temperature (298 K) and compared with those of raw p-SiC and pure ZIF-8 (Figure 5). At *P* = 100 kPa, pure ZIF-8 exhibited a CO_2_ uptake of 28.64 cm^3^ g^−1^ _STP_, whereas the ZIF-8/p-SiC composite showed a CO_2_ uptake of 21.61 cm^3^ g^−1^ _STP_, significantly higher than raw p-SiC (0.79 cm^3^ g^−1^ _STP_). This indicates (i) efficient utilization of ZIF-8 within the composite and (ii) retention of ZIF-8 adsorption performance after incorporation. Compared with previously reported ZIF-8-based composites [27,32], the ZIF-8/p-SiC composite exhibits comparable CO_2_ adsorption capacity, while the p-SiC substrate offers relative advantages in terms of tunable pore structure and mechanical strength.

Under equivalent experimental conditions (298 K), N_2_ adsorption was also assessed. The ZIF-8/p-SiC composite exhibits negligible N_2_ adsorption at room temperature (Figure 5), which is attributed to the combined influence of pore size and the affinity of the imidazolate linkers toward CO_2_, determining ZIF-8′s CO_2_/N_2_ adsorption selectivity [33]. Specifically, CO_2_ has a smaller kinetic diameter (3.3 Å) and higher diffusivity, whereas the diffusion of N_2_ (3.64 Å) within the ZIF-8 pore channels (3.4 Å) is kinetically more restricted, and its interaction with the framework is relatively weaker under ambient conditions [34]. Additionally, the amine groups within the imidazolate linkers of ZIF-8 can interact with CO_2_ molecules through Lewis acid-base interactions, thereby providing specific adsorption sites favorable for CO_2_ adsorption [35]. Overall, the hierarchical pore structure of the ZIF-8/p-SiC composite enhances molecular transport and improves the accessibility of adsorption sites through physisorption-dominated mechanisms, thereby endowing the composite with superior CO_2_ adsorption performance.

## 4. Conclusions

In this study, we employed a flexible and scalable synthetic strategy to successfully grow ZIF-8 nanocrystals in situ within a p-SiC substrate, constructing a ZIF-8/p-SiC composite featuring a hierarchical pore structure. The robust three-dimensional porous framework of p-SiC provides both mechanical support and transport channels for the nucleation and growth of ZIF-8, enabling uniform distribution and stable anchoring of MOF crystals throughout the macropore network. Concurrently, the intrinsic microporous structure and high specific surface area of ZIF-8 endow the composite with enhanced CO_2_ adsorption capacity, as well as superior molecular sieving and selective transport properties. Furthermore, the low-cost industrial-grade p-SiC and mild ZIF-8 synthesis conditions confer potential for large-scale production of ZIF-8/p-SiC composites in terms of economic feasibility, reproducibility, and scalability.

Significantly, the developed ZIF-8/p-SiC composite holds promise as an ideal porous medium for investigating MOF-rock interactions and CO_2_ geological sequestration mechanisms, offering valuable insights for the design of functionalized MOF/porous media tailored for subsurface reservoir engineering.

## Figures and Tables

**Table 1:** Zeta potential of raw p-SiC and pretreated p-SiC.

Samples	Zeta Potential (mV)
Raw p-SiC	−25.30 ± 2.75 mV
Pretreated p-SiC	−48.60 ± 1.20 mV

**Table 2:** Elemental composition of the ZIF-8/p-SiC composite determined by EDS.

Element	Weight Percentage (wt%)
C	33.70
N	7.16
O	22.91
Si	33.02
Zn	3.21

**Table 3:** Pore parameters of raw p-SiC, pure ZIF-8, and ZIF-8/p-SiC composite. a: BJH method; b: DR method.

Samples	*S*_BET_ (m^2^ g^−1^)	*V*_total_ (cm^3^ g^−1^)	*D*_p_ (nm)
Raw p-SiC	0.1	0.012 ^a^	6.15 ^a^
Pure ZIF-8	1588	0.938 ^b^	-
ZIF-8/p-SiC	18.3	0.021 ^a^	3.28 ^a^

## Data Availability

The original contributions presented in this study are included in the article. Further inquiries can be directed to the corresponding author.
